# Prevalence and Predictors of Willingness to Make Advance Directives among Macao Chinese

**DOI:** 10.3390/ijerph18157942

**Published:** 2021-07-27

**Authors:** Sok Man Leong, Kuai In Tam, Sok Leng Che, Ming Xia Zhu

**Affiliations:** 1 Education Department, Kiang Wu Nursing College of Macau, Macao SAR, China; zmx@kwnc.edu.mo; 2 Research Management and Development Department, Kiang Wu Nursing College of Macau, Macao SAR, China; joannatam@kwnc.edu.mo; 3 Nursing and Health Education Research Centre, Kiang Wu Nursing College of Macau, Macao SAR, China; shirley@kwnc.edu.mo

**Keywords:** advance directives, end-of-life care, general public, Macao, Chinese

## Abstract

While advance directives (ADs) are considered to be part of government’s healthcare agenda, there has not been any public consultation for legislation, nor investigation regarding the public’s views about ADs in the Chinese culture of Macao. The current study explored the Macao Chinese people’s willingness to make ADs. Data were collected from 724 residents aged 18 years and above. Results showed that 533 respondents (73.6%) claimed that they were willing to complete an AD if the document was recognized legally. The experience of caring for relatives or friends with terminal illnesses, palliative care as the preferred end-of-life treatment option, and scoring higher in the Hospice Care Attitude Scale were the predictors of willingness to make ADs. Results of the study suggested that there was a relatively high number of people who would consider setting up ADs. Our study recommends that healthcare professionals should equip themselves to raise ADs-related discussions with patients. Moreover, the Macao Government is responsible for facilitating the introduction and implementation of ADs in order to improve overall end-of-life care quality in Macao.

## 1. Introduction

The prevalence of chronic diseases and the probability of disability increase with age, and most people may rely on medical and nursing care for an extended period of time before death. Death and dying is inevitable, and end-of-life (EoL) situations are often more complex for people with multiple long-term illnesses. Despite the medical complexity involved, many patients are not equipped and often failed to express their medical wishes in the terminal phase. As a result, family members are often being given the responsibility of making treatment decisions for the patients. However, under the influence of filial piety in the Chinese culture, in which children have the moral and even legal obligation to care for their parents financially, physically, and psychologically [1,2], adult children often struggle to forgo life-prolonging treatments for their parents, in order to fulfill the obligation of filial piety [3]. The insistence on excessive, often meaningless, treatments not only leads to unnecessary suffering for the dying patients, but has also put a huge strain on medical services.

To respect individuals’ dignity and to deliver medical care that is aligned with individuals’ values and care preferences, planning documents such as advance directives (ADs) are available for individuals to voice and record care preferences while they are still conscious and have decisional capacity [4]. ADs have been formalized as a legal document in a number of developed countries such as the United States, the United Kingdom, Australia, and Switzerland. Despite the ADs’ legal validity, the completion rate among the general population varies in different countries; approximately 40.0% of the general public has completed ADs in the United States [5], while only 14.0% have completed them in Australia [6]; only 24.0% of adults aged 55 and above have completed an AD in Switzerland [7]. In Chinese communities, Taiwan is the first region to establish ADs-related legislations; the introduction of the Hospice Palliative Care Act (Natural Death Act) was established in 2000 and the Patient Right to Autonomy Act was implemented in 2019 [8]. Moreover, Hong Kong first introduced the concept of ADs back in 2004, and it was subsequently implemented in a non-official manner in 2006. After years of public engagement, a second public consultation was introduced in 2019 in order to further review the public’s readiness for ADs [9]. Although many Asian regions have not established ADs-related legislation, the demand for the right to make one’s own treatment decision is evidently on the rise. Previous studies showed that more than half of the older Korean adults agreed to writing ADs [10,11], and 60.9% of Hong Kong adults would prefer to make an AD if legislated [12]. In terms of predictors of willingness to make ADs, Chung et al. [12] found that people who had heard of do-not-attempt-cardiopulmonary-resuscitation (DNACR) were more willing to make ADs. Another study found that people who had considered what life-sustaining treatments entailed supported the legalization of ADs, and those who wanted to be informed about their own medical status were more likely to agree with writing ADs [11]. With respect to inhibitors, being female and students were found to have significantly reduced one’s willingness to make ADs [12,13]. Furthermore, people with a low level of education were less likely to approve ADs [7].

In Macao, the average life expectancy has not stopped increasing, and it has reached 83.4 years in 2017 [14]. The proportion of adults aged 65 or above will exceed 20.0% in 2031 according to the prediction of the Statistics and Census Service of Macao Special Administrative Region Government [15]. Despite end-of-life care becoming a major public health issue in Macao’s ageing society, there is still no ADs-related legislation in Macao. At present, doctors and patients, or their families, can sign an agreement stating the decision to reject resuscitation or other life-sustaining treatments. However, the agreement is based primarily on a hospital’s informed consent policy, and the document provided is conceptually different from ADs. Albeit members of the Life Science Ethics Committee have reached consensus that ADs should be implemented in Macao [16], and there has been increasing emphasis on care for people at the end of their lives [17], the discussion about implementing ADs has not continued and the introduction of ADs has come to a halt. 

To date, ADs are considered to be part of government’s healthcare agenda, but there is a significant lack of information reflecting Macao residents’ views on ADs. Existing studies found that some socio-demographic factors, medical status, and knowledge of life-sustaining treatments would influence people’s willingness to make ADs [7,10,11,12,13]. However, research on people’s willingness to set up ADs is limited in Chinese communities. Hence, focusing on the Chinese cultural context of Macao, the current study aimed to explore the Macao Chinese residents’ willingness to make ADs, and to identify the predictors of their willingness to make ADs. The research questions of this study were as follows: (1) What is the prevalence of willingness to make ADs among the Macao Chinese residents? (2) What are the predictors of willingness to make ADs among the Macao Chinese residents?

## 2. Materials and Methods

### 2.1. Study Design and Participants

The current study was a cross-sectional survey conducted in Macao from July to September 2020. Referencing the population statistics at the end of 2018 in Macao [18], non-probability quota sampling according to age groups was employed. Participants who (1) were Macao residents aged 18 or above, and (2) could understand informed consent materials and questionnaire content were included in this study. Ethical approval was obtained from a higher education institution in Macao. According to the sample size calculation for survey study [19,20], the minimal sample size for this study was 384, with a level of significance of 0.05, absolute error of 5%, and at type 1 error of 5%. 

### 2.2. Instrument and Data Collection

The structured questionnaire was written in Chinese and formulated by the research team based on related literature [12,21,22]. The questionnaire had four sections: (1) Socio-demographic data: including age, sex, education level, occupation, income, marital status, any children, religious belief, and experiences of caring for relatives or friends with terminally illness: “yes” or “no”; (2) Self-rated health: measured on a 5-point scale from “very bad” to “very good”; (3) Preferences of EoL treatment and attitudes towards EoL care: including the question “If the doctor has diagnosed you with an incurable illness, and you are estimated to have less than 6 months to live, which of the followings would you choose?” Respondents were presented with four choices: (i) “I will accept all life-prolonging interventions, despite discomfort or sufferings may occur during the treatment process”; (ii) “I will accept treatment interventions that can ease pain and suffering, or alleviate discomfort caused by symptoms, despite my life may not be extended”; (iii) “I don’t know/I don’t have a decision”; and (iv) “Don’t want to answer.” The Hospice Care Attitude Scale was used to assess participants’ attitudes towards EoL care. The scale was validated by the research team, with the Cronbach’s α coefficient of 0.79, test–retest reliability was 0.94, the Item–Content Validity Index of 1.00 and a good level of Construct Validity, which demonstrated good reliability and validity, and detailed information was reported in another paper [23]. The Hospice Care Attitude Scale contained 11 items, measured on a 5-point scale from “Very unimportant” to “Very important.” Total score of the scale ranges from 11 to 55; the higher the score, the more important hospice care means for the respondents at the EoL; (4) Willingness to make an AD: “Would you complete an AD to express your medical wishes of EoL care if the document is legally recognized?” and “What are the reasons for not willing to complete the AD document?” (this question was only asked if the participants chose not to make an AD, and some possible reasons were provided for respondents to choose from). Another question asked was, “Do you think that it is necessary to discuss your wishes about EoL treatments and nursing care with medical professionals?”

The questionnaire was reviewed by three experts in the field of palliative and EoL care from Beijing, Hong Kong, and Macao. The three experts agreed that the questionnaire was primarily suitable to address the enquiry of this study. The survey was pretested in 55 Macao residents aged 18 or above to check the readability of the revised questionnaire. The respondents expressed that they could understand the content and it took an average of 10 to 15 min to complete the entire questionnaire. For individuals aged between 18 and 59 interested in participating in this study, self-administered electronic questionnaire was distributed through social media platforms (such as Facebook and WeChat groups of the research team and their friends), and two of the largest social organizations in Macao (one was Macao Federation of Trade Unions, its membership composed of people from different industries; and the other was the Macao General Association of Neighborhood, which had more than 40,000 members) recruited adults from different backgrounds across various age groups. The questionnaire was hosted on Survey Monkey (https://www.surveymonkey.com/, accessed on 15 July 2020) online platform. Residents aged 60 or above could join as a member in any of the community center for older adults in Macao. Thus, considering the relatively lower level of education and the lack of familiarity to online survey, the research team decided to recruit individuals aged 60 and above from community centers for older adults in Macao, and those centers were under three major social service organizations. The staff of the centers assisted in promoting the research, and those who were interested would be interviewed in a private area in those centers. Verbal informed consent was obtained from the participants before the interviews, in which participants were assisted by a trained interviewer to complete the questionnaire face-to-face. The study received a total of 1001 responses of which 724 were valid, with the completion rate of 72.3%. Of the valid responses, 86.2% were self-administered online, 13.8% were completed face-to-face by interviewers.

### 2.3. Statistical Analysis

Data analyses were conducted using the software SPSS 22.0. Descriptive statistics were performed to summarize the responses of the participant. Chi-square or *t*-tests were used to examine the associations of socio-demographic characteristics, caring experiences, self-rated health status, preference of EoL treatments, scores of the Hospice Care Attitude Scale, and the willingness of making ADs. Multiple logistic regression analysis was conducted by using the willingness of making ADs as dependent variable, and other items mentioned in the questionnaire as independent variables to identify predictors. Only significant variables in correlation analysis were used as independent variables in logistic regression with enter method, and the Hosmer–Lemeshow test was used to test the goodness of fit of the model (*χ*^2^ = 12.97, *p* = 0.11). All *p*-values less than 0.05 were considered statistically significant. Since some respondents did not provide an answer for “education level” and “average monthly income,” only 677 responses could be used for regression analysis.

## 3. Results

### 3.1. Demographic Characteristics

The study received a total of 724 valid responses; 64.9% of the respondents were female. In summary, the age distribution of the participants was similar to the population of Macao; 57.2% of the respondents had college level or higher education; 66.6% of respondents were married or cohabited; 74.2% respondents were employed or self-employed, and 24.2% of those were working as professionals. Over 40.0% of the respondents rated their health as good or very good, and 38.5% of the respondents had cared for relatives or friends suffering from terminal illnesses (Table 1).

### 3.2. Willingness to Make Advance Directives

Results showed that 533 respondents (73.6%) claimed that they would complete an AD if the document was recognized legally, 50 respondents (6.9%) would not complete an AD, and 141 respondents (19.5%) did not know or undecided. For those who were unwilling to set up ADs, the top reason found in this study was because they did not know the specific content of ADs (54.5%), followed by they did not know the procedures of setting up ADs (42.4%), and the respondents were afraid that they might change their mind after setting up ADs (41.4%; Table 2). Furthermore, a high number of respondents (*n* = 619, 85.5%) expressed that it was necessary to discuss the treatment and care at the end of their lives with healthcare professionals.

### 3.3. Predictors of Willingness to Make Advance Directives

Age, educational level, marital status, average monthly income in the past year, caring experiences, EoL treatment option, and the score of the Hospice Care Attitude Scale were found to be significantly correlated with willingness to make ADs by using Crosstab or *t*-test analyses (Table 3).

The results of multiple logistic regression showed that caring experiences, EoL treatment option, and the score of the Hospice Care Attitude Scale were the predictors of willingness to make ADs. The respondents who had cared for relatives or friends with terminal illnesses were more willing to make ADs than those who had not (odds ratio [OR] = 1.68, 95% confidence interval [CI] [1.14, 2.49]). The respondents who chose suffering-alleviating treatments, despite knowing that their limited lives might not be extended, were more likely to set up ADs than those who chose life-prolonging treatments (OR = 2.20, 95% CI [1.53, 3.17]). The respondents who scored higher in the Hospice Care Attitude Scale were more willing to make ADs (OR = 1.06, 95% CI [1.02, 1.10]). All the other factors were not statistically significant (Table 4).

## 4. Discussion

The results of this Macao-based study showed that more than 70.0% of our participants were willing to make ADs if the document is legally recognized; the percentage of willingness was similar with previous studies conducted in other Asia regions [10,11,12]. Existing studies show that there is a relatively high number of Asian people who would consider making ADs. Our study found that the lack of information about ADs was the main reason preventing people from being willing to make ADs. Such results are also reflected in other Hong Kong-based studies in which people’s knowledge on ADs was found to be inadequate, and has prevented them from making ADs [12,13]. As Jang et al.’s [24] study affirmed, people who had a higher level of ADs-related knowledge were more likely to complete ADs, illuminating that raising public awareness and understanding of ADs is necessary in its promotion and implementation.

In spite of the inadequate understanding of ADs, our study found that most respondents believed it was necessary to discuss the treatment and care at the end of their lives with healthcare professionals. This finding is supported by Kwok et al. [25] in that patients agreed that it was helpful to have ADs discussion with healthcare professionals, as it can help to lessen their pressure in making EoL-related decisions. Alano et al.’s study [26] found that the probability of completing ADs was related to healthcare providers’ willingness to initiate ADs’ discussion and explaining ADs to their patients and families. It is found that nurses also agreed that they should facilitate patients to complete ADs [27]. Despite healthcare professionals believing that they have the responsibility to initiate ADs discussions, there are a number of factors preventing such discussions from taking place: (i) the lack of ADs knowledge among healthcare professionals [28]; (ii) the lack of experience and confidence in nurses to initiate ADs; and most importantly, (iii) the absence of ADs-related legislation [27,29]. Similar to Chung et al.’s [12] study, our study affirmed that the general population considered ADs a good approach to make advance decisions if they were diagnosed to be terminally ill, and people also thought that ADs could help to relieve suffering at the end of their lives [26], indicating a high demand from the public to be involved in their EoL care decision making. However, the absence of legislation and the lack of discussion in the community has prevented the overall implementation of ADs in Macao. Our study suggested that there is certainly a need for public education in order to promote people’s knowledge regarding ADs, thereby enhancing the public support for establishing ADs-related legislation. Considering neighboring cities, such as Hong Kong, which has had a long and challenging journey toward introducing ADs, a recent study in Hong Kong found that over 70.0% of the respondents believed the promotion of ADs was still not enough [13]. Thus, the Macao Government should urgently address the need for public promotion, and enforce continuous education for healthcare professionals, equipping them to raise ADs-related discussions with patients in the Chinese cultural society of Macao. 

In terms of predictors of willingness to make ADs, our findings were consistent with Lee et al.’s study [10] that participants who had cared for relatives or friends with terminal illnesses had significantly increased chances of making ADs. Our study suggested that respondents who had witnessed other people’s suffering at the end of their lives would be more likely to understand the difficulties in making EoL treatment decisions. As suggested by Tang et al. [30], carers were reported to feel guilty and helpless after making EoL decisions for their loved ones, and carers were also uncertain about the choices they had made. This may explain why our study respondents decided to make ADs for themselves, as they might have been subjected to guilt and helplessness in making decisions for people they had once cared for. Respondents would choose to make advance decisions for themselves in order to avoid being a burden for their carers. Such a phenomenon is supported by Park and Song’s [11] study in that authors found the main reason encouraging Korean older adults to make ADs was that they did not want to become a burden for their family.

Our study suggested that respondents who preferred palliative care as their EoL treatment option, and those who scored higher in the Hospice Care Attitude Scale were more willing to make ADs. Findings of our study are in line with existing evidence that positive attitude towards EoL care and advance decision making is associated with the willingness to complete ADs [31]. Furthermore, previous studies have affirmed that the people’s willingness to make ADs is related to their wish to be in control of their own care, and the right to refuse futile treatments [25,32]. Moreover, older patients with ADs were less likely to receive futile life-sustaining treatments, such as cardiopulmonary resuscitation, intubation, and mechanical ventilation, during the last months of their lives [33]. Our findings illuminated that for people who preferred palliative care as their EoL treatment option, they would also demand for the right to make advance decisions for themselves. Taking into consideration of the study findings and the Chinese society of Macao, it is evident that early public education on ADs would benefit patients who would like to make ADs but do not have adequate knowledge supporting them to do so. Besides, legislation for ADs is needed in order to empower people who would like to make their advance decisions and to support carers of the terminally ill. Healthcare professionals should assess patients’ preference of EoL treatment option and initiate ADs-related discussion with patients and their carers. As suggested by Powers [34], the promotion and education of ADs should be integrated into the public health approach, in order to strengthen public engagement on issues relating to EoL care and ADs; future studies should, therefore, seek to find effective ways and strategies to stimulate ADs’ discussion, thereby transforming people’s intentions into actions. 

Similar to Ko et al.’s [31] findings, standard demographic variables, such as age, sex, educational level, and marital status, were not the predictors of the willingness to make ADs among general population in Macao, while some studies found that being female [12] and students [13] were the inhibiting factors, and old age [26,35,36], a high level of education [10,37], and a low economic status were the promoting factors [10], our study did not observe such relationships. Further exploration on the impact of demographic variables on the willingness of making ADs is necessary.

### Limitations

Non-probabilistic quota sampling was employed in order to ensure the age distribution of participants was similar to the Macao population. However, the proportion of female and professional participants in this study were higher than the proportion of the Macao population, and hence sample representativeness may be restricted. Nevertheless, females were more willing to participate in research as observed in another study [12] in which random sampling method was used. In addition, the respondents in this study covered every occupation category according to Statistics and Census Service of Macao, which may lessen the bias. Moreover, the respondents’ voluntary participation in this study may reflect their relatively positive attitudes towards EoL care issues. Potential bias could occur as the research has adopted both online and face-to-face interviews to collect data. Our study primarily collected data from the online survey; this may be related to the higher education and professional occupation tendencies of our participants. Future studies should take this limitation into consideration.

## 5. Conclusions

Being the first study investigating the public’s willingness to make ADs in Macao, results of our study indicate an urgent need for healthcare professionals to equip themselves to engage in ADs discussions with patients and their families. Moreover, the Macao Government and clinical settings should act to facilitate the introduction and implementation of ADs. Furthermore, our study suggests that there was a relatively high number of people who would consider setting up ADs, but the lack of understanding of ADs was the main inhibitors deterring people’s willingness to make ADs. Preference of palliative care as EoL treatment option and caring experience of the terminally ill were the main predictors of willingness to make ADs. To improve the quality of EoL care, the Macao Government should have a specific work plan to launch public education, and review current policies in order to prepare for ADs legislation. Furthermore, our findings also serve as a reference for other Asian regions since public education and promotion on ADs is still limited in Southeast Asia and Mainland China. Meanwhile, EoL care is becoming a major public health concern due to the rapidly ageing population; local governments should be aware of the public’s view on ADs, and equip healthcare professionals promptly in order to provide EoL care that can fulfill people’s wishes.

## Figures and Tables

**Table 1:** Socio-demographic characteristics of participants (*n* = 724).

Variable	*n*	%
Sex		
Male	254	35.1
Female	470	64.9
Age (year)		
18–39	305	42.1
40–64	299	41.3
≥65	120	16.6
Education		
Primary school or below	94	13.0
Secondary school	211	29.1
Bachelor or above	414	57.2
No answer	5	0.7
Marital status		
Not married	150	20.7
Married/cohabited	482	66.6
Separated/divorced	38	5.2
Widowed	54	7.5
Children		
Have children	511	70.6
No children	213	29.4
Religious belief		
No religion	418	57.7
Christianity	120	16.6
Buddhism/Chinese folk beliefs	186	25.7
Occupation		
Professional	175	24.2
Medical (assistant) professional	86	11.9
Technician	106	14.6
Disciplined services	40	5.5
Attendant	130	18.0
Not employed	187	25.8
Average monthly income (Macao pataca)		
<9999	147	20.3
10,000–19,999	135	18.6
20,000–29,999	176	24.3
≥30,000	224	30.9
No answer	42	5.8
Caring experiences		
Yes	279	38.5
No	445	61.5
Self-rated health		
Good	301	41.6
Not good	423	58.4

**Table 2:** Reasons for not setting up advance directives (*n* = 191).

Item	*n*	%
I don’t know the specific content of advance directives	104	54.5
I don’t know the procedures of setting up advance directives	81	42.4
I am afraid that I might change my mind	79	41.4
I worry I might cause trouble for other people	46	24.1
I worry I might not get the healthcare and care I need	37	19.4
Others	19	9.9
I am too young now; it is not necessary	15	7.9
I don’t want to answer	12	6.3

**Table 3:** Correlations of willingness to make advance directives by socio-demographic characteristics and attitude of hospice care (*n* = 724).

Variables	No/Don’t Know/Not Decided		Yes		*χ*^2^/*t*	*p* Value
	n/M	%/SD	n/M	%/SD		
Sex					0.50	0.54
Male	63	24.8	191	75.2		
Female	128	27.2	342	72.8		
Age (year)					7.94	*0.02*
18–39	76	24.9	229	75.1		
40–64	71	23.7	228	76.3		
≥65	44	36.7	76	63.3		
Education (*n* = 719)					15.53	*0.00*
Primary school or below	36	38.3	58	61.7		
Secondary school	67	31.8	144	68.2		
Bachelor or above	88	21.3	326	78.7		
Marital status					11.82	*0.01*
Not married	43	28.7	107	71.3		
Married/cohabited	117	24.3	365	75.7		
Separated/divorced	7	18.4	31	81.6		
Widowed	24	44.4	30	55.6		
Children					0.92	0.34
Have children	140	27.4	371	72.6		
No children	51	23.9	162	76.1		
Religious belief					2.72	0.26
No religion	109	26.1	309	73.9		
Christianity	26	21.7	94	78.3		
Buddhist/Chinese folk beliefs	56	30.1	130	69.9		
Occupation					10.60	0.06
Professional	45	25.7	130	74.3		
Medical (assistant) professional	17	19.8	69	80.2		
Technician	31	29.2	75	70.8		
Disciplined services	9	22.5	31	77.5		
Attendant	26	20.0	104	80.0		
Not employed	63	33.7	124	66.3		
Average monthly income (Macao pataca) (*n* = 682)					14.50	*0.00*
<9999	57	38.8	90	61.2		
10,000–19,999	34	25.2	101	74.8		
20,000–29,999	44	25.0	132	75.0		
≥30,000	48	21.4	176	78.6		
Caring experiences					8.28	*0.00*
Yes	57	20.4	222	79.6		
No	134	30.1	311	69.9		
Self-rated health					3.81	0.06
Not good	123	29.1	300	70.9		
Good/very good	68	22.6	233	77.4		
Treatment option (*n* = 716)					18.45	*0.00*
Life prolongation/don’t know/not decided	94	35.5	171	64.5		
Suffering alleviation	94	20.8	357	79.2		
Attitude of hospice care	45.85	4.6	47.65	5.2	−4.48	*0.01*

Note: *p* value in italics mean *p* < 0.05.

**Table 4:** Multiple logistic regression models of willingness to make advance directives (0 = no/don’t know/not decided; 1 = yes) (*n* = 677).

Variables	*B*	SE	Wald	*p* Value	Exp (*B*)	95% CI for Exp (*B*)	
						Lower	Upper
Constant	−3.36	1.03	10.70	*0.00*	0.03		
Age (year) (ref.:18–39)							
40–64	0.08	0.23	0.12	0.73	1.08	0.69	1.70
≥65	0.45	0.50	0.79	0.37	1.57	0.58	4.19
Education (ref.: Primary school or below)							
Secondary school	−0.13	0.49	0.07	0.79	0.88	0.34	2.28
Bachelor or above	0.38	0.53	0.52	0.47	1.47	0.52	4.18
Marital status (ref.: Not married)							
Married/cohabited	0.31	0.25	1.51	0.22	1.36	0.83	2.21
Separated/divorced	0.47	0.50	0.87	0.35	1.59	0.60	4.24
Widowed	−0.39	0.45	0.75	0.39	0.67	0.28	1.64
Average monthly income (Macao pataca) (ref.: <9999)							
10,000–19,999	0.56	0.34	2.71	0.10	1.76	0.90	3.43
20,000–29,999	0.49	0.33	2.28	0.13	1.64	0.86	3.11
≥30,000	0.62	0.34	3.42	0.06	1.87	0.96	3.62
Cared for relatives/friends (ref.: no)	0.52	0.20	6.82	*0.01*	1.68	1.14	2.49
Treatment option (ref.: life prolongation)	0.79	0.19	17.87	*0.00*	2.20	1.53	3.17
Attitude of hospice care	0.06	0.02	11.00	*0.00*	1.06	1.02	1.10

Note: *p* value in italics mean *p* < 0.05.

## Data Availability

Data available on request due to ethical considerations. The data presented in this study are available on request from the corresponding author.

## References

[1] Liu B.S., Huang H.C. (2009). Family care for the elderly and the importance of filial piety. J. Nurs..

[2] Standing Committee of the National People’s Congress Law of the People’s Republic of China on Protection of the Rights and Interests of the Elderly. http://www.lawinfochina.com/display.aspx?lib=law&id=1159&CGid=.

[3] Li L.B. (2013). Clinical review: Ethics and end-of-life care for critically ill patients in China. Crit. Care.

[4] The Hospital Authority of Hong Kong “Advance Care Planning”? “Advance Directives”? Do not attempt “Cardiopulmonary Resuscitation”? Know More for Patients and Their Family Members. https://www.ha.org.hk/haho/ho/psrm/Public_education1.pdf.

[5] Rao J.K., Andersen L.A., Lin F., Laux J.P. (2014). Completion of advance directives among U.S. consumers. Am. J. Prev. Med..

[6] White B., Tilse C., Wilson J., Rosenman L., Strub T., Feeney R., Silvester W. (2014). Prevalence and predictors of advance directives in Australia. Intern. Med. J..

[7] Vilpert S., Borrat-Besson C., Maurer J., Borasio G.D. (2018). Awareness, approval and completion of advance directives in older adults in Switzerland. Swiss Mid. Wkly..

[8] Laws and Regulations Database of the Republic of China Patient Right to Autonomy Act. https://law.moj.gov.tw/ENG/LawClass/LawAll.aspx?pcode=L0020189.

[9] Legislative Council of the Hong Kong Special Administrative Region of China Advance Healthcare Directives of Patients. https://www.legco.gov.hk/research-publications/english/essentials-1819ise07-advance-healthcare-directives-of-patients.htm.

[10] Lee J.E., Shin D.W., Son K.Y., Park H.J., Lim J.Y., Song M.S., Park Y.H., Cho B. (2018). Factors influencing attitudes toward advance directives in Korean older adults. Arch. Gerontol. Geriatr..

[11] Park J., Song J.A. (2015). Predictors of agreement with writing advance directives among older Korean adults. J. Transcult. Nurs..

[12] Chung R.Y.N., Wong E.L.Y., Kiang N., Chau P.Y.K., Lau J.Y.C., Wong S.Y.S., Yeoh E.K., Woo J.W. (2017). Knowledge, attitudes, and preferences of advance decisions, end-of-life care, and place of care and death in Hong Kong. A population-based telephong survey of 1067 adults. J. Am. Med. Dir. Assoc..

[13] Chan C.W.H., Wong M.M.H., Choi K.C., Chan H.Y.L., Chow A.Y.M., Lo R.S.K., Sham M.M.K. (2019). Prevalence, perception, and predictors of advance directives among Hong Kong Chinese: A population-based survey. Int. J. Environ. Res. Public Health.

[14] Statistics and Census Service of Macao Special Administrative Region Government Macao Data 2017. http://www.dsec.gov.mo/CMSPages/c_mn_indicator.aspx.

[15] Statistics and Census Service of Macao Special Administrative Region Government (2014). Trends and Challenges of Population Ageing.

[16] Health Bureau of Macao The Second Working Meeting of Life Science Ethics Committee in 2019. https://www.gov.mo/zh-hant/news/310049/.

[17] Macao SAR Government (2016). Ten Year Action Plan of Elderly Service of Macao.

[18] Statistics and Census Service of Macao Special Administrative Region Government (2019). Yearbook of Statistics 2018.

[19] Sharma S.K., Mudgal S.K., Thakur K., Gaur R. (2020). How to calculate sample size for observational and experimental nursing research studies?. Natl. J. Physiol. Pharm. Pharmacol..

[20] Charan J., Biswas T. (2013). How to calculate sample size for different study designs in medical research?. Indian J Psychol. Med..

[21] Hsu C.P., Chen H.W., Lee S.Y., Tsou M.T. (2012). Knowledge and attitude toward hospice palliative care among community-dwelling aged Taiwanese—Analysis of related factors. Int. J. Gerontol..

[22] Mcllfatrick S., Hasson F., McLaughlin D., Johnston G., Roulston A., Rutherford L., Noble H., Kelly S., Craig A., Kernohan W.G. (2013). Public awareness and attitudes toward palliative care in Northern Ireland. BMC Palliat. Care.

[23] Che S.L., Tam K.I., Zhu M.X., Leong S.M. (2021). Revision and validation of the hospice care attitude scale. Macau J. Nurs..

[24] Jang Y., Park N.S., Chiriboga D.A., Radhakrishnan K., Kim M.T. (2017). The knowing-doing gap in advance directives in Asian Americans: The role of education and acculturation. Am. J. Hosp. Palliat. Med..

[25] Kwok Y.L., Leung W.M., Agarwal A., Wong K.H. (2018). Advance directives in Chinese patients with cancer: A retrospective study in a single palliative care unit. J. Pain Manag..

[26] Alano G.J., Pekmezaris R., Tai J.Y., Hussain M.J., Jeune J., Loius B., El-Kass G., Ashraf M.S., Reddy R., Lesser M. (2010). Factors influencing older adults to complete advance directives. Palliat. Support. Care.

[27] Lo K.C. (2020). A study of Macao Nurses on the Correlations between Knowledge, Attitude, Confidence and Experience with Advance Directives. Master’s Thesis.

[28] Peicius E., Blazeviciene A., Kaminskas R. (2017). Are advance directives helpful for good end of life decision making: A cross sectional survey of health professionals. BMC Med. Ethics.

[29] Coleman A.M.E. (2012). Physician attitudes toward advanced directives: A literature review of variables impacting on physicians attitude toward advanced directives. Am. J. Hosp. Palliat. Med..

[30] Tang C.S.H., Lam L.C.W., Chiu H.F.K. (2007). Attitudes to end-of-life decisions: A survey of elderly Chinese with dementia and their carers. Asian J. Gerontol. Geriatr..

[31] Ko E., Lee J., Hong Y. (2016). Willingness to complete advance directives among low-income older adults living in the USA. Health Soc. Care Community.

[32] Genewick J.E., Lipski D.M., Schupack K.M., Buffington A.L.H. (2018). Characteristics of patients with existing advance directives: Evaluating motivations around advance care planning. Am. J. Hosp. Palliat. Med..

[33] Yen Y.F., Huang L.Y., Hu H.Y., Sun W.J., Ko M.C., Lee Y.L., Huang S.J., Chu D. (2018). Association of advance directives completion with the utilization of life-sustaining treatments during the end-of–life care in older patients. J. Pain Symptom Manag..

[34] Powers J.S. (2020). Promoting advance directive discussions. Palliat. Med. Hosp. Care Open J..

[35] Chu D., Yen Y.F., Hu H.Y., Lai Y.J., Sun W.J., Ko M.C., Huang L.Y., Chen C.C., Curtic J.R., Lee Y.L. (2018). Factors associated with advance directives completion among patients with advanced care planning communication in Taipei, Taiwan. PLoS ONE.

[36] Zhang R.J., Fu Y., Xiang Q.F., Yang M., Chen L., Shi Y.K., Yu C.H., Li J.Y. (2016). Knowledge, attitudes, and influencing factors of cancer patients toward approving advance directives in China. Support. Care Cancer.

[37] Lovell A., Yates P. (2014). Advance care planning in palliative care: A systematic literature review of the contextual factors influencing its uptake 2008–2012. Palliat. Med..

